# Green Synthesis of BPL-NiONPs Using Leaf Extract of *Berberis pachyacantha*: Characterization and Multiple In Vitro Biological Applications

**DOI:** 10.3390/molecules27072064

**Published:** 2022-03-23

**Authors:** Siraj Uddin, Javed Iqbal, Luqman Bin Safdar, Saleem Ahmad, Banzeer Ahsan Abbasi, Raffaele Capasso, Mohsin Kazi, Umar Masood Quraihi

**Affiliations:** 1 Department of Plant Sciences, Faculty of Biological Sciences, Quaid-i-Azam University, Islamabad 45320, Pakistan; usiraj85@gmail.com; 2 Department of Botany, Bacha Khan University, Charsadda 24420, Khyber Pakhtunkhwa, Pakistan; banzeer.abbasi@bs.qau.edu.pk; 3 School of Biosciences, Sutton Bonington Campus, University of Nottingham, Sutton Bonington, Leicestershire LE12 5RD, UK; luqman.safdar@nottingham.ac.uk; 4 School of Agriculture, Food and Wine, Waite Research Institute, University of Adelaide, Glen Osmond, SA 5064, Australia; 5 Guangdong Provincial Key Laboratory for Breast Cancer Diagnosis and Treatment, Shantou University Medical College, Shantou 515041, China; ahmad.chilas@gmail.com; 6 Department of Agricultural Sciences, University of Naples Federico II, 80055 Portici, Naples, Italy; rafcapas@unina.it; 7 Department of Pharmaceutics, College of Pharmacy, King Saud University, P.O. Box 2457, Riyadh 11451, Saudi Arabia; mkazi@ksu.edu.sa

**Keywords:** *Berberis pachyacantha*, leaf extract, green synthesis, BPL-NiONPs, antioxidant, antimicrobial, cytotoxicity, phytotoxicity, nano fertilizer

## Abstract

An eco-friendly biogenic method for the synthesis of nickel oxide nanoparticles (NiONPs) using phytochemically rich *Berberis pachyacantha* leaf extract (BPL) was established. To achieve this purpose, 80 mL of BPL extract was used as a suitable reducing and capping agent for the synthesis of NiONPs. The synthesis of BPL-based nickel oxide nanoparticles (BPL@NiONPs) was confirmed using different microscopic and spectroscopic techniques: UV Visible spectroscopy (UV-Vis), Fourier-transform infrared spectroscopy (FTIR), X-ray diffraction (XRD), energy dispersive X-ray (EDX), dynamic light scattering (DLS) and scanning electron microscopy (SEM) analysis. Spectroscopically, BPL-NiONPs was found with a pure elemental composition (oxygen and nickel), average size (22.53 nm) and rhombohedral structure with multiple functional groups (-OH group and Ni-O formation) on their surface. In the next step, the BPL extract and BPL@NiONPs were further investigated for various biological activities. As compared to BPL extract, BPL@NiONPs exhibited strong biological activities. BPL@NiONPs showed remarkable antioxidant activities in terms of 2,2-diphenyl-1-picrylhydrazyl (76.08%) and total antioxidant capacity (68.74%). Antibacterial action was found against *Pseudomonas aeruginosa* (27 mm), *Staphylococcus aureus* (20 mm) and *Escherichia coli* (19.67 mm) at 500 µg/mL. While antifungal potentials were shown against *Alternaria alternata* (81.25%), *Fusarium oxysporum* (42.86%) and *Aspergillus niger* (42%) at 1000 µg/mL. Similarly, dose-dependent cytotoxicity response was confirmed against brine shrimp with IC50 value (45.08 µg/mL). Additionally, BPL@NiONPs exhibited stimulatory efficacy by enhancing seed germination rate at low concentrations (31.25 and 62.5 µg/mL). In conclusion, this study depicted that BPL extract has important phytochemicals with remarkable antioxidant activities, which successfully reduced and stabilized the BPL@NiONPs. The overall result of this study suggested that BPL@NiONPs could be used as nanomedicines and nanofertilizers in biomedical and agrarian fields.

## 1. Introduction

Nanotechnology is one of the most outstanding and fast-growing areas of science, which has the ability to make particles at the nano scale (1 and 100 nm) in at least one dimension [1]. Nanotechnologies make the nanoparticles (NPs) more inspiring concerning their physicochemical and biological properties as compared to bulk ingredients [2]. Due to their nano size and high surface-to-volume ratio, the synthesized NPs have great demands in multiple fields, such as the biomedical, pharmaceutical, industrial, commercial, mechanical, electrical, agricultural, and environmental fields [2,3]. Nanoparticles have been reported with potent biological application against various infectious diseases as the causative agents of many infectious diseases are continuously developing resistance against available drugs such as pesticides, fungicides and other synthetic chemical compounds [4]. Therefore, an alternative, easy and safe option is the use of natural products with potent biomedical potential against these causative agents [5]. Moreover, with the arrival of nanotechnology, researchers are developing nanoparticles (NPs) to control the growth of diverse infectious pathogens [6].

Nanoparticles are synthesized by physical, chemical, and biological approaches. Among these, biological approaches using medicinal plants are gaining great popularity due to their cheap, easy and eco-friendly behaviours [7]. Through green synthesis, various metal and metal oxide NPs such as gold (Au), silver (Ag), iron (II) oxide (FeO), zinc oxide (ZnO), cobalt (II) oxide (CoO) and nickel oxide (NiO) have been prepared and used for numerous purposes [3,8,9,10].

The green synthesis of nanoparticles uses plant extracts and precursor salts (nitrates, sulphates, oxides, and chloride) as strong reducing, stabilising and capping agents [1]. Plants contain many phytochemicals in the form of secondary metabolites, which act as reducing agents to convert metal ions into metal atoms, while salt has electron donating and reduction potential. These two factors are involved in increasing the electron density. As a result, metals in the ionic form are easily separated from the anionic part and are finally reduced and stabilized by plant extracts. Plant extracts have numerous phytocompounds such as alkaloids, polyphenols, flavonoids, terpenoids, sugar, and proteins, which reduce the metal ions into a stable state. Mostly, functional groups such as the hydroxyl group (-OH) of biomolecules are involved in nanoparticle formation [2]. However, the size, structure and morphological nature of NPs depend upon the bioactive phytochemicals present in the plant extract [11]. Recently, green synthesised NiONPs have received great admiration due to their easy, cost-effective and biological applications against various pathogens [3,12]. Numerous biological applications (cytotoxic, antimicrobial, anti-inflammatory and anticancer) and environmental potentials (pollutant and dye degradations) of NiONPs have been reported [13,14,15]. The biological activities of NiONPs, such as antimicrobial and antioxidant activities, can play a significant role in biomedical fields and nutraceutical industries to make useful medicines, surgical apparatus, preserved food additives and other edible products [1]. Due to the nano size, the nanoparticles displayed potent biological activities against various infectious pathogens. Nanoparticles have more attachment and penetration ability with the cell membrane of pathogens as compared to bulk materials [3]. Different studies have shown that the inhibitory potential of nanoparticles is due to the penetration and interference of nanoparticles with intracellular machinery. Briefly, this particle released nickel ions that attached and penetrated inside the cells and caused leakage of the cell membrane. Inside the cell, the nickel oxide nanoparticles generated reactive oxygen species (ROS), which are toxic, highly reactive by-products of oxygen metabolism such as peroxides, hydroxyl radicals, superoxides and singlet oxygen [4]. ROS directly inhibited the cellular life machinery such as breaking phosphate and hydrogen bonding of the DNA strand, destroying the three-dimensional structure of proteins and causing oxidative stress in the powerhouse of the cell [5].

For better growth and development, the plant needs various minerals and fertilizers. Different types of fertilizer are available on the market, all of which play a significant role during stress conditions by enhancing the nutritional status of autotrophs. Globally, in the past several decades, chemical fertilizers have been extensively used to increase the quality and quantity of plant products. However, these types of synthetic fertilizer are not recommended economically and environmentally due to their various problems such as being costly, their unavailability, leaching, environment pollution, etc. [6]. Consequently, the best, easy alternative method is the adaptation of green nano technology to limit the loss of crop yields by producing nano-fertilizers [7]. Recently, a new era of research, ‘the process of nano fertilization’ has been gaining great attention globally. Khalaki and Moameri [16] encapsulate the positive effect of NPs on critical stages of plants and described that NPs have great stimulatory effects on the seed germination and seedling growth of the plants. Some metal and metal oxide nanoparticles such as silicon dioxide (SiO_2_), iron (II) oxide (FeO), copper (II) oxide (CuO), ceric oxide (CeO_2_), zinc oxide (ZnO) and *Titanium dioxide* (TiO_2_) have been reported with stimulatory effects on plant life cycles [17,18]. In the present study, the eco-friendly green method was adopted to synthesise NiO nanoparticles from precursor salt (nickel nitrate) using aqueous leaf extracts of Berberis pachyacantha (family Berberidaceae) [19]. *Berberis pachyacantha* is widely distributed in the temperate and semi-temperate areas of Pakistan, such as the north-west Himalayas and Kashmir [19]. The plants of this family are famous for medicinal properties due to the presence of bioactive phytocompounds in its fruit, bark and roots [20]. Limited literature is available on the phytochemicals and biological applications of *B. pachycantha*. However, various species of this family have been used by local communities for the treatment of various illness and diseases such as high blood pressure, blood purification, poisoning that can be caused by a snake bite, cough, diabetes, microbial infection, and internal wounds of human beings and livestock [21,22]. Recently, 18–37 compounds have been identified from *B. crataegina*, *B. integerrima*, *B. aetnensis* and *B. libanotica* [8]. Similarly, other *Berberis* species such as *B. Balochistanica*, *B*. *aristata*, *B. orthobotrys*, *B. thomsonian*, *B. vulgaris*, *B. asiatica*, *B. croatica* and *B. thomsoniana* have an adequate amount of phenol and flavonoid content [9,10,11,12,13,14,15,16]. These reported phytochemicals have a rich pool of electrons, which are directly involved in radical scavenging activities against biotic and abiotic stresses [17,18]. Oxidative stresses are responsible for numerous health conditions, including cardiovascular diseases, neurodegenerative disorders and most deadly diseases such as cancers [19]. The potent antioxidant activity of *Berberis* plants could be due to the presence of different phenolic and flavonoid contents that absorbed and neutralized the free radicals [20,21,22].

These biomolecules play a great role in reducing and capping metal salts into NPs. Previously, different species such as *B. valgarus* and *B. aristate* have been used for the biofabrication of Ag NPs and Zn ONPs [23,24,25,26]. To the best of our knowledge and according to a literature review, this is the first study to provide information about the biochemical, antioxidant and biological potentials of leaf extracts of a BP plant. After the confirmation of biomolecules in a leaf extract of BP, this broth was sourced as a reducing and capping agent during the green synthesis of NiONPs. The synthesized BPL-NiONPs were characterized using different analytical methods (UV, FTIR, XRD, EDS, SEM and DLS), followed by multiple in vitro biological activities.

## 2. Results

### 2.1. Physical Characterization of BPL Extract

#### 2.1.1. UV-VIS Analysis

**Figure 1:**
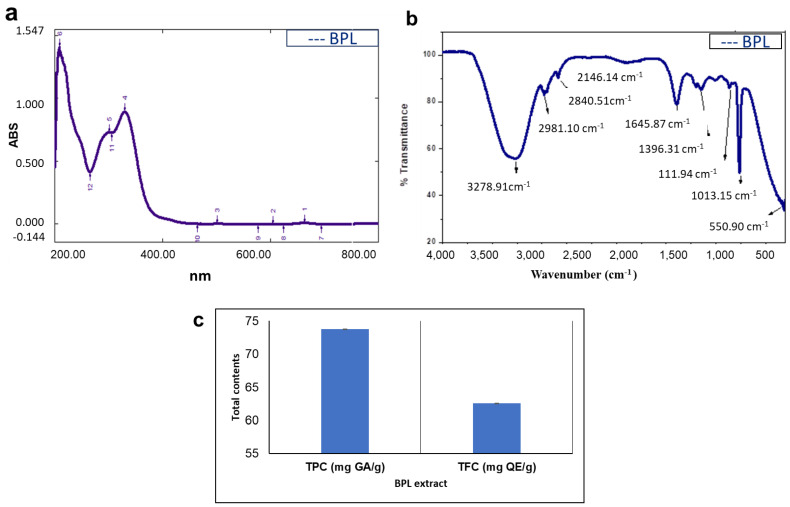
Spectroscopic and phytochemical profile of BPL extract (**a**) UV-Visible spectroscopy (**b**) FTIR analysis (**c**) TPC and TFC.

The UV-VIS profile of BPL broth was characterized using the range of 200 to 800 nm. BPL extract showed multiple peaks at 209 nm, 265.5 nm, 301, 306 nm, 330 nm and 663 nm, with absorption at 1.41, 0.414, 0.73, 0.725, 0.892 and 0.008, respectively (Figure 1a).

#### 2.1.2. FTIR Analysis

The extracts were subjected to FTIR to separate and identify the functional groups of the molecules present in plant samples based on peak values. The spectrum profile and peak values representing functional groups were compared with the IR standard chart, as shown in Figure 1b and Table 1. The analysed part showed comparable peak values and confirmed the presence of phenols, alcohols, amides, amines, alkanes, alkenes, aromatic compounds, carboxylic acid and alkyl halides. Most interestingly, the absence of peaks at 2220–2260 cm^−1^ showed that there were no cyanide derivatives in the BPL extract.

### 2.2. Phytochemical Analysis of BPL Extract

#### Total Phenolic and Flavonoid Contents

A significant amount of total phenolic content and total flavonoid content were shown by BPL extraction (Figure 1c). The TPC was articulated as gallic acid equivalents (milligram of gallic acid per gram of extract), while the TFC of the BPL extract was enumerated as Quercetin equivalents (milligram of Quercetin per gram of plant extract). Comparatively more TPC (73.74 mg GA/g) was detected compared to TFC (62.58 mg QE/g).

### 2.3. Physical Characterization of BPL-NiONPs

**Figure 2:**
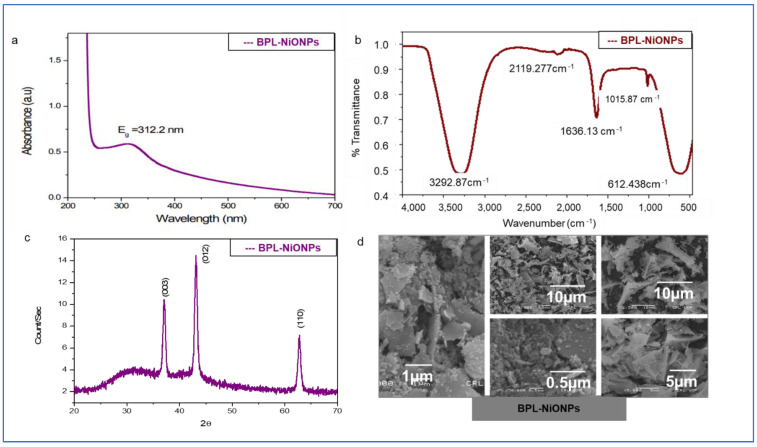
Spectroscopic analysis of BPL-NiONPs: (**a**) UV-Visible spectroscopy; (**b**) FTIR analysis; (**c**) XRD analysis; (**d**) SEM image (X1000, X5000, X2500, X10,000, X30,000).

**Figure 3:**
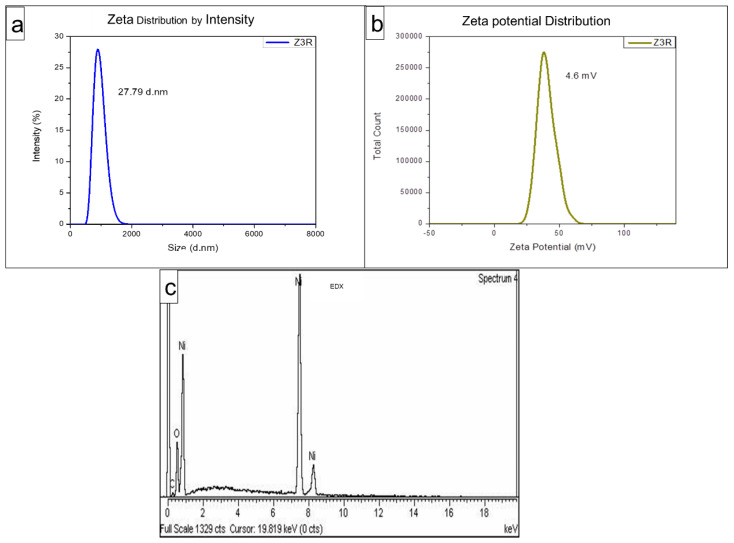
Zeta size distribution (**a**), zeta potential (**b**), and EDS analysis (**c**) of BPL-NiONPs.

The reduction of nickel nitrate into NiONPs by BPL extract can be verified by the change in colour from green to dark grey. The UV-Vis profile of BPL-NiONPs exhibited the characteristic absorption peak which was noticed at 312.2 nm (Figure 2a). The presence of functional groups on the surface of BPL-NiONPs was determined by FTIR analysis. Figure 2b presents the multiple absorption bands associated with functional groups, such as 612.48 cm^−1^ (Ni-O), 1015.87 cm^−1^(C-O) 1636.13 cm^−1^ (C=N) and 3292.87 cm^−1^(OH), respectively. These functional groups depicted the presence of phytochemicals such as phenols, alcohols, amides, amines, alkanes and carboxylic acid, which bind to the surface of nickel oxide nanoparticles and maintained their stability. Further, the structural nature of the BPL-NiONPs was investigated by XRD analysis and a pure crystalline edifice was confirmed. The planes of the peaks were (003), (012), and (110) at 0.2411 nm, 0.2088 nm, and 0.147 nm, respectively (Figure 2c). Additionally, the average size of BPL@NiONPs was ~22.53 nm and the peaks were matched with standard JCPDS Card #: ICSD ID 00-022-1189, as shown in Table 2. The peaks sharpness and intensity showed that BPL-NiONPs are synthesized in BPL extract. Moreover, the XRD spectra also confirmed the purity of BPL-NiONPs as no other peaks were detected. The zeta size distribution and ζ- potential of the synthesized BPL-NiONPs was confirmed by DLS. The hydrodynamic distribution and ζ- potential of BPL-NiONPs were 27.79 nm and 4.6 mV, as shown in Figure 3a,b. The DLS result confirmed that our NPs are less aggregated in colloidal medium. The morphological profile of BPL@NiONPs was performed using SEM analysis (Figure 2d). Additionally, the purity and the presence of Ni-O was confirmed by EDX analysis (Figure 3c).

### 2.4. Comparative Biological Analysis of BPL Extract and BPL-NiONPs

#### 2.4.1. Antioxidant Potential

**Figure 4:**
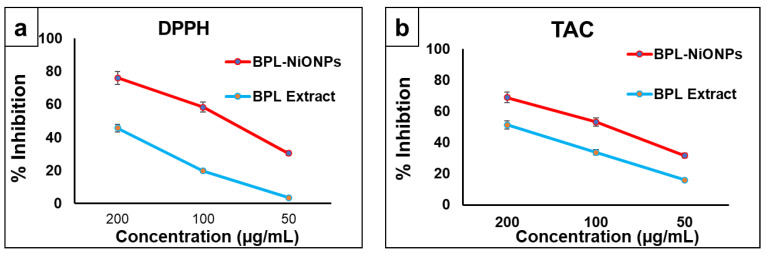
Antioxidant activity of BPL and BPL-NiONPs: (**a**) DPPH analysis; (**b**) TAC analysis. Obtained data are presented as mean ± standard deviation (*n* = 3).

The results confirmed that both BPL extract and BPL-NiONPs have dose-dependent antioxidant activities in terms of DPPH and TAC. Notably, BPL-NiONPs have a higher % inhibition of DPPH radicals (76.08–30.48%) compared to BPL extract (45.72–3.53%). Similarly, efficient TAC activity was shown by BPL-NIONPs (68.74–31.52%) relative to BPL extract (51.18–15.90%) across different concentrations (200–50 µg/mL). In short, strong DPPH scavenging activity and moderate TAC activity was reported in both substituents (Figure 4).

#### 2.4.2. Antimicrobial Potential of BPL Extract and BPL-NiONPs

**Figure 5:**
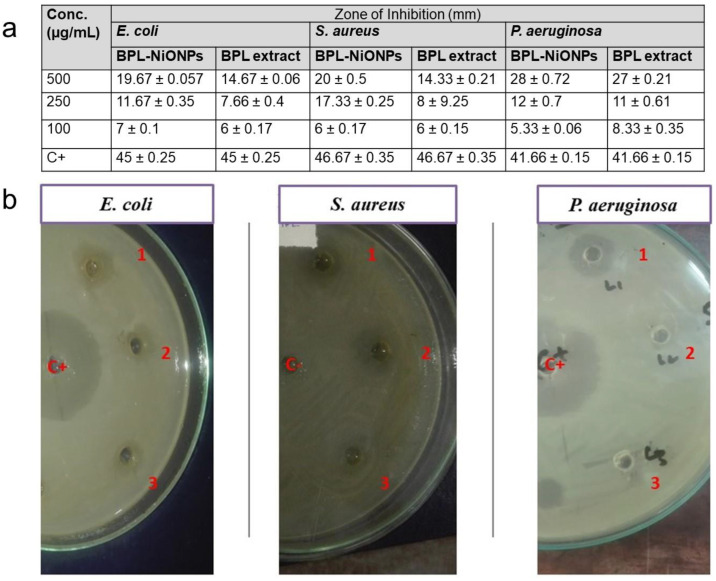
Antibacterial activity of BPL-NiONPs against *E. coli*, *S. aureus and P. aeruginosa.* (**a**) Zone of inhibition against 500, 250 and 100 µg/mL; (**b**) The clear inhibited area of tested pathogens at 500, 250 and 100 µg/mL of BPL-NiONPs. The obtained data are presented as mean ± standard deviation (*n* = 3). The numbers: 1, 2 and 3 represent the concentration of BPL@NiONPs (500, 250 and 100 µg/mL), while C+ and C− refer to the positive (Kanamycin) and negative control (distilled water), respectively.

In the present study, the antibacterial actions of BPL extract and BPL@NiONPs were tested as potential antibacterial agents using three bacterial strains: *E. coli*, *S. aureus* and *P. aeruginosa.* Both representatives showed dose-dependent antibacterial response against all three bacterial pathogens, as depicted in Figure 5a. Comparatively, synthesized BPL-NiONPs showed potent bacterial inhibition as compared to BPL extract. The maximum zone of inhibition of BPL-NiONPs was observed against *P. aeruginosa* (28 mm) and the minimum zone of inhibition was detected against *E. coli* (19.67 mm) at 500 µg/mL (Figure 5b).

#### 2.4.3. Mycelial Growth in Inhibition

**Figure 6:**
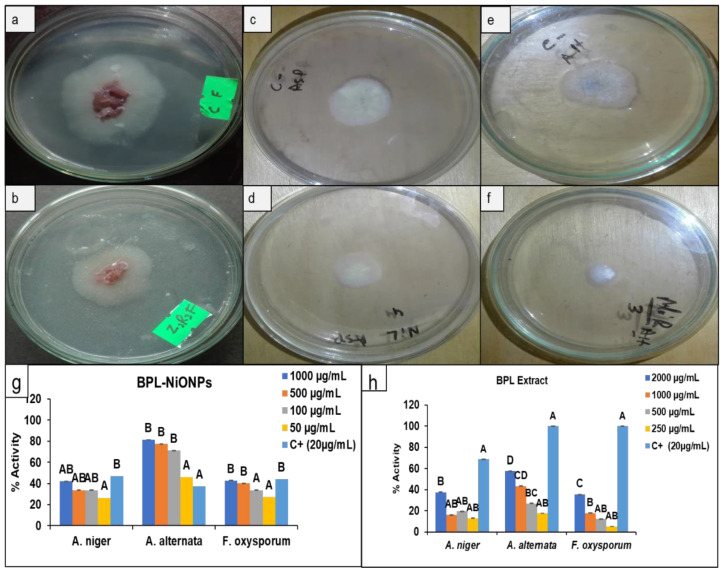
Antifungal analysis of *Berberis pachyacantha* leaf extract (BPL) BPL-NiONPs at 1000 µg/mL against *F. oxysporum*: (**a**) control (non-treated) and (**b**) treated with BPL-NiONPs; *A. niger*: (**c**) control (non-treated) and (**d**) treated with BPL-NiONPs; and *A. alternata*: (**e**) control (non-treated) and (**f**) treated with BPL-NiONPs. Antifungal activity: (**g**) BPL extract; (**h**) BPL-NiONPs. C+ refers to positive control (fluconazole). The obtained data are presented as mean ± standard deviation (*n* = 3). Letters specify a significant difference (*p* < 0.05) between control and BPL-NiONPs treated samples.

The food poison method (FPM) was used to observe the effect of BPL extract and BPL-NiONPs on the mycelium growth of three fungal pathogens including *Aspergillus niger*, *Alternaria alternate* and *Fusarium oxysporum*, whereas fluconazole was used as the positive control. A dose-dependent response was exhibited by both constituents against all analysed pathogens. In the case of BPL extract, the maximum colonial growth inhibition was carried out against *A. alternata* (57.65%), followed by *A. niger* (37.70%) and *F. oxysporum* (35.71%) at 2000 µg/mL (Figure 6h), while, on the other hand, the BPL-NiONPs showed more inhibitory activity at 1000 µg/mL against all three pathogens as compared to the BPL extract. The BPL-NiONPs showed maximum inhibition against *A. alternata* (81.25%), followed by *F. oxysporum* (42.86%) and *A. niger* (42.17%) at 1000 µg/mL (Figure 6g). In short, *A. alternata* was found more susceptible towards both BPL-NiONPs and BPL extract (Figure 6).

#### 2.4.4. Cytotoxic Activity

**Figure 7:**
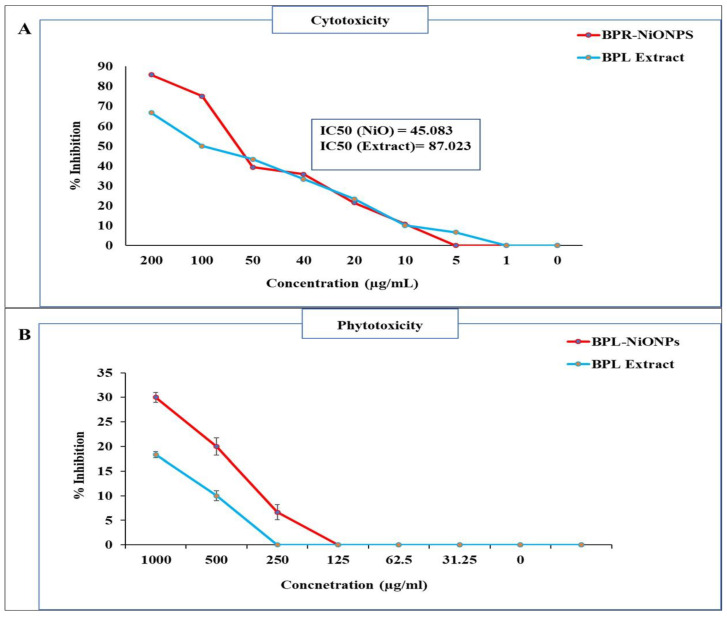
Cytotoxicity and phytotoxicity assays of BPL and BPL-NiONPs: (**A**) brine shrimp mortality test; (**B**) relative germination inhibition (%) of radish seeds. Obtained data are presented as mean ± standard deviation (*n* = 3).

To determine the cytotoxic nature of BPL extract and BPL-NiONPs, an in vitro lethality test was conducted using brine shrimp. Both solutions showed a dose-dependent cytotoxic response against brine shrimp at different concentrations (Figure 7A). Considerable cytotoxic activities were proven in BPL extract and BPL-NiONPs with IC_50_ values of 87.023 and 45.083 µg/mL, respectively. However, the green BPL@NiONPs showed more mortality (85.71%) compared to the extract (66.67%) at 200 µg/mL. These findings indicate that BPL extracts have bioactive compounds that are potent against brine shrimps and play a significant role in the biofabrication of NPs.

#### 2.4.5. Stimulatory and Inhibitory Potential

**Figure 8:**
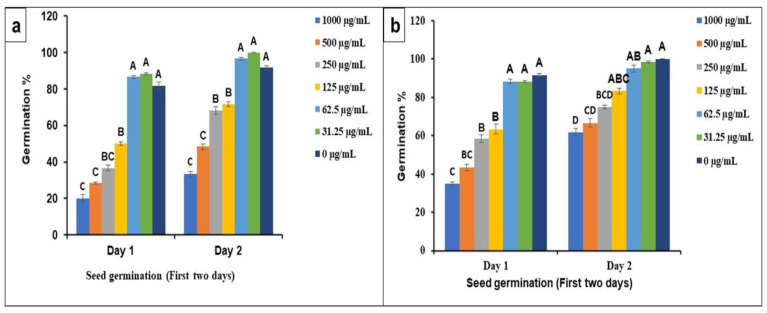
Phytotoxicity and stimulatory effect of BPL extract and BPL-based NiONPs on relative germination rate (RGR) of seed during first two days (D1 and D2): (**a**) rate of germination of radish seeds against various concentrations (1000–31.25 µg/mL) of BPL-NiONPs; (**b**) rate of germination of radish seeds against various concentrations (1000–31.25 µg/mL) of BPL extract. The obtained data are presented as mean ± standard deviation (*n* = 3). Letters specify a significant difference (*p* < 0.05) between control and BPL-NiONP-treated samples.

Figure 8a,b, indicate the seed germination rate at applied concentrations (1000–31.25 µg/mL) of BPL extract and BPL-NiONPs. The seed germination was stimulated at lower concentrations (31.25 and 62.5 µg/mL), while it was delayed at higher concentrations (250, 500 and 1000 µg/mL). At lower concentrations (31.25 and 62.5 µg/mL), the speed of germination was enhanced 7% and 5% more by BPL-NiONPs than non-treated controls during first day of counting. Similar results were reported for the second day (Day 2) of counting by increasing the germination speed up to 8.3% and 5% at 31.25 and 62.5 µg/mL, respectively. Further germination percentage (%) was calculated and no inhibitory effect at a lower dose was reported. However, the inhibition of seed germination (6.67, 20 and 30%) was observed at higher concentrations (Figure 7B). In brief, BPL-NiONPs showed stimulatory activity at lower concentrations by increasing the germination rate without any inhibitory effect. Aside from BPL-NiONPs, BPL extracts have no satisfactory effects on seed germination.

## 3. Discussion

### 3.1. Analysis of BPL Extracts

In recent years, medicinal plants have been gaining the attention of pharmacological industries due to the presence of bioactive compounds that have no/fewer side effects and easy availability [25]. Medicinal plants are a rich source of secondary metabolites with significant biological activities and are also used as dietary and nutrient supplements [18]. In the present study, for the first time, BPL extract was found as a highly rich source of TPC and TFC. These reported phytochemicals have a pool of electrons that are directly involved in radical scavenging activities against biotic and abiotic stresses [17]. A similar result was reported in the leaf extract of *B. thunbergini* [26]. The BPL extract was further characterized using UV-Visible and FTIR spectroscopy, and various peaks were detected. The UV-Vis result of the present study showed that leaf extracts exhibited peak values in the range of 209 nm, 265.5 nm, 301 nm, 306 nm, 330 nm and 663 nm, respectively. Usually, it is reported that the peaks in the ranges of 240–280 nm, 290–350 nm, 300–380 nm and 600–700 nm indicated the presence of glycosides, phenolic acids, flavones and chlorophyll contents [21]. Similarly, in another study it was reported that the presence of one or multiple peaks in the range of 200–400 nm clearly indicated the presence of unsaturated hydrocarbon groups and heteroatoms [22]. Similarly, the FTIR result in Table 2 shows various functional group stretchings, which interpret the relevant compounds present in the tested extract. The obtained functional groups specified the following biomolecules; phenols, alcohols, amides, amines, alkanes, alkenes, aromatic compounds, carboxylic acid and alkyl halides [23]. The peak values of the BPL extract are comparable with previous studies in which similar peaks were detected in the stem and leaves of the *B. aristata* plant [24]. The results of the present study show that BPL extract has the best antioxidant activities in terms of DPPH and total antioxidant capacity. This present finding is strongly supported by the results of other studies that *Berberis* species, such as *B. baluchitanica* [13] and *B*. *lyceum* [27], have strong antioxidant activities. Similar findings with high antioxidant activity have been reported by Malik, et al. [28], using *Citrus nobilis* peel methanolic extract. These remarkable activities might be due to the kinetic release of antioxidants from plant extract [28,29]. Based on the results of TPC, TFC, FTIR spectra and potent antioxidant activity, BPL extract was used further for the synthesis of NiONPs.

### 3.2. Analysis of Green Synthesized BPL-NiONPs

#### 3.2.1. Spectroscopic Screening of BPL-NiONPs

The FTIR spectrum of BPL-NiONPs displayed multiple vibrations representing various functional groups, such as OH, C-H, C=C, CHO, and C-O stretching, which clearly revealed the information about NiO in NiNO_3_. Similar IR bands have been reported in the range of 470 and 800 cm^−1^ for Ni-O vibration [30,31,32]. The FTIR results are strongly supported by the XRD and EDS profiles of BPL-NiONPs, where no impurity was detected. The XRD profile of BPL-NiONPs showed an average size of 22.53 nm and a rhombohedral shape with respect to Debye Scherer’s equation and JCPDS Card #: ICSD ID 00-022-1189. Present XRD analyses bear resemblance to previous studies of NiONPs using various plant extracts [30,33]. The pure nature of BPL@NiONPs was confirmed by EDS analysis and the presence of nickel, oxygen and trace amounts of carbon were reported. The occurrence of carbon in the spectra might be due to the attachment of functional groups of phytochemicals on the surface of NPs and attributed to grid assistance [3,30]. The enormity of zeta potential represented the stability of the particles; for example, NPs with high zeta potential should have high stability and a low agglomeration of NPs. In the present data, the zeta potential and hydrodynamic size distributions of BPL@NiONPs were 4.6 mV and 27.79 nm. These results are a sign of stability and low aggregation in colloidal suspension, as previous studies have also described NPs with charges in the range of −25 and +25 mV as a sign of stability [34]. Our finding agrees with earlier reported data about NiONPs using *Rhamnus virgate*, *Rhamnus triquetra* and *Rhamnella gilgitica* plants [30,35].

#### 3.2.2. Biological Analysis of BPL-NiONPs

Owing to their nano nature and biological activities, along with recent developments in medicine, the application of NPs against infectious diseases is becoming a hot topic for researchers. The typical potential of NiONPs coupled with a nano size, high surface are and high energy make them a desirable candidate for various biological activities. The results of the present study exhibited that BPL@NiONPs showed strong DPPH scavenging activity and TAC activity as compared to BPL extracts. Subsequently, the antioxidant potencies of BBS-NiONPs might be due to the presence of phytocompounds in the BPL extract, which interact with the surface of NPs [31,33]. In the present study, the dose-reliant antibacterial activity was reported against all three pathogens. Comparatively, synthesized BPL-NiONPs showed potent bacterial inhibition as compared to the BPL extract. The maximum zone of inhibition of BPL-NiONPs was observed against *P. aeruginosa* (28 mm) and the minimum zone of inhibition was detected against *E. coli* (19.67 mm) at 1000 µg/mL. The small size and high surface-to-volume ratio make the NPs more inspiring concerning their biological properties as compared to bulk ingredients [36]. Generally, the bacterial mortality depends on the quantity of NPs, methods of synthesis and treatment duration [37]. A similar finding was reported against some microbial pathogens such as *S. typhimurium*, *S. aureus*, *E. coli* and *K. pneumonia* using copper nanoparticles capped with 1% gum arabic [38]. The significant antimicrobial activities of BPL-NiONPs may be due to the presence of various bioactive functional groups and phenolic compounds of BPL extract, which individually also showed remarkable antimicrobial activities. The results of the present study were in accordance with the findings of Malik, Najda, Bains, Nurzyńska-Wierdak and Chawla [28] who observed the antimicrobial effect of Citrus nobilis peels against pathogenic bacteria and fungi.

**Figure 9:**
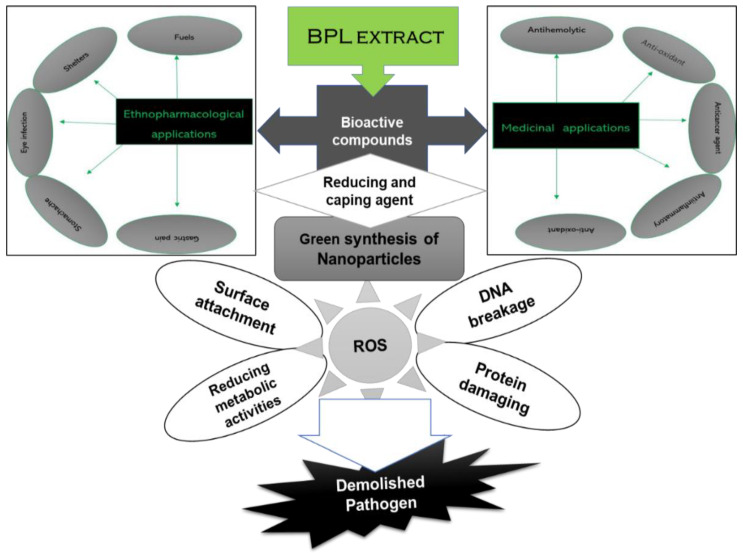
Applications of Berberis plant in biomedical and green synthesis of NPs and the hypothetical action mechanism of nanoparticles against microbial pathogens.

The BPL-NiONPs showed maximum inhibition against *A. alternata* > *F. oxysporum* > *A. niger* at 1000 µg/mL. The mycelial inhibition of *A. alternata* and *F. oxysporum* at 1 mg/mL was also reported in previous studies using iron oxide nanoparticles [3,39]. In recent times, researchers have effectively used NiONPs as an antifungal agent against several fungal pathogens, including *M. racemosus*, *R. solani*, *A*. *flavus*, *A*. *niger*, *C*. *albican*, and *F*. *solanai* [33,40]. The antimicrobial activities of the nanoparticles are still under dispute. However, the rudimentary mechanism of inhibition might be due the reduction of nanoparticles by bioactive compounds. As a result, interaction between released ions from NPs and the cell membrane of microbes occurred. This interaction leads to penetration inside the cell and starts the inhibition of metabolic machinery, and, ultimately, destruction of the cell ensues [3,30,41] (Figure 9). The inhibitory frequency of NPs varies due to different factors, including salt, biogenic source, species of pathogen, shape, size, and concentration, of the nanoparticles used [41]. The results of the present study show that BPL-NiONPs are not only useful for the control of phytopathogens but also used as alternative to chemical fungicides. The strong cytotoxic potential of leaf part and their synthesized NPs were confirmed by brine shrimp mortality test. Both carcasses showed cytotoxic response with IC_50_ values 45.083 (BPL-NiONPs) and 87.023 µg/mL (BPL extract), respectively. However, biogenic BPL-NiONPs showed more mortality (85.71%) as compared to extracts (66.67%) at 200 µg/mL. Research studies have concluded that nanoparticles show cytotoxic potentials by reducing metabolic activities, generating reactive oxygen species (ROS), and damaging DNA and protein.

These findings indicate that BPL extract has bioactive compounds that are potent against brine shrimp larvae and play a significant role in the biofabrication of NPs. The present result has agreement with the results of previous study of Iqbal, Abbasi, Mahmood, Hameed, Munir and Kanwal [31] as dose-dependent repressive reactions were observed during biological activities by BPL-NiONPs. Literature review has shown that biological activities of NPs may possibly be due to the crystalline nature and nano scale size of the particles and the reducing nature of the extract [3]. Cytotoxicity effect depends upon the concentration of NiONPs, which makes NIONPs applicable in various fields, especially in toxin removal and biomedical fields [42].

#### 3.2.3. Stimulatory Effect and Phytotoxic Effect

Plants have a significant contribution as a primary producer in the food web of all ecosystems. Plants also exchange different element with biotic and abiotic components of the environment. For better growth and development, the plant needs various minerals and fertilizer, which are not easily available due to complex formation, leaching, degradation by hydrolysis, photolysis and parsing of the elements. Consequently, the best easy, alternative method is the adaptation of green nano technology to limit the loss of crop yields by producing nano fertilizer. However, these NPs have positive or negative influences on the life processes of plants.

Nowadays, many studies have reported that nanomaterials have both positive and negative effects on living organisms and the environment. However, these dual effects are totally dependent on concentration, size, stability, shape, coating of the NPs and behaviours of the organism toward that NPs [43]. Plants also showed different behaviours towards various NPs. In numerous studies, it has been reported that NPs can penetrate, transform, and be translocated to various parts of the plant. Szőllősi et al. [44] extensively summarized the stimulatory and inhibitory effects of different NMs. They found that stimulatory effects might be due to an increase in antioxidant and metabolic activities at the cellular level, while inhibitory effects of different NPs on seed germination and seedling growth might be due to chromosomal aberration, abnormalities in cell division, hormonal imbalance and over production of ROS. Khalaki, et al. [45] encapsulate the positive effect of NPs on the critical stage of plant and described that NPs have great stimulatory effects on seed germination and seedling growth of the plants. Some metal and metal oxide nanoparticles are Ag, Au, SiO_2_, FeO, CuO, CeO_2_, ZnO and TiO_2_, which have been reported to have stimulatory effects on plant life cycles [46,47]. These NPs play a great role in agricultural fields by increasing the germination rate, breaking the dormancy, and limiting the usage of chemical fertilizers [48,49]. The most critical stages in a plant’s life are seed germination and seedling growth. The current study is innovative by reporting the nano fertilizing nature of BPL@NIONPs. At lower concentrations, BPL@NIONPs increased the germination rate with no side effects on the final germination of seeds. The stimulatory mechanism might be due the interaction of NPs with the seed coat, releasing ions, enhancing the nutrient and water uptake. All these factors help in breaking the seed dormancy and the germination of seedlings [48,50,51,52]. Hence, BPL-NiONPs can be used as a plant growth promoter, nano fertilizer and an alternative to chemical fertilizers. The BPL-NiONPs showed potent biological potential as compared to plant extract. This might be due to the nano size with single dimension (1 and 100 nm) of the nanomaterials, which make the NPs more inspiring concerning their physicochemical properties as compared to bulk ingredients. Careful screening is required as diverse responses have been shown by different plants toward applied NPs [53]. Therefore, it is important to consider the positive role of nanomaterials, but also focus on their downsides ahead of release in the agriculture market.

## 4. Materials and Methods

### 4.1. Berberis pachyancantha (BP)

#### 4.1.1. Plant Collection

The medicinal plant *Berberis pachyancantha* (BP) was collected during April-June 2018–2019 from the mountainous regions of Kaghan valley Narran, District Mansehra, Khyber Pakhtunkhwa, Pakistan. After sampling, the plant was identified and deposited (RAW-100433) in the National Herbarium, Islamabad, Pakistan. The plant leaves were carefully separated, washed and properly dried at room temperature for two weeks.

#### 4.1.2. *Berberis pachyancantha* Leaf (BPL) Extracts Preparation

The leaf extract of *Berberis pachyacantha* was extracted and evaluated for various phytochemicals and biological activities. The dry leaves were properly ground into a fine powder, and about 20 g of powder was added into 200 mL of distilled water. The solution of the extract was incubated for 2 h at 80 °C in a water bath with proper stirring, yielding a dark yellow-colour extract with pH 5.3. After cooling, the BPL extract was passed through muslin cloth and was then filtered three times (Whatman filter paper), and the solvent was evaporated using a rotary evaporator (BUCHI Rotavapor R-220, Shanghai, China). The obtained dried powder was kept at 4 °C for further analysis

### 4.2. Green Synthesis of BPL-NiONPs

#### 4.2.1. The BPL Assisted Synthesis of NiONPs

To determine the presence of biomolecules in BPL extract, characterization of aqueous extract (BPL) was carried out through UV-visible and FTIR spectroscopy. The leaf extract showed enough phytochemicals and important functional groups with the best antioxidant activities. After confirmation of the presence of bioactive compounds in BPL, the dried pure extract of BPL was re-dissolved in 100 mL distilled water for synthesis of BPL-NiONPs. Up to 80 mL of BPL extract was found as a suitable capping agent for synthesis of NiONPs. The BPL-mediated NiONPs were prepared by mixing nickel nitrate (1 gm) with BPL (80 mL) extract and subjected to heating at 70 °C for 90 min with appropriate stirring. During heating and stirring, the homogenous mixture changed colour (green to dark grey), which formerly indicating the synthesis of BPL@NiONPs. The BPL@NiONPs solution was centrifuged at 10,000 rpm for 10 min and supernatant was discarded. The collected pellet of BPL@NiONPs was washed with distilled water and incubated at ~100 °C for 3 h to evaporate the remaining water molecules. For effective crystallization, the dried sample was annealed for 4 h at 200 °C using an air furnace (KSL-1100X, MTI Corporation, Hefei, China). Finally, the annealed powder of BPL-assisted NiONPs was stored at room temperature for further characterization and biological application.

#### 4.2.2. Characterization of BPL-NiONPs 

**Figure 10:**
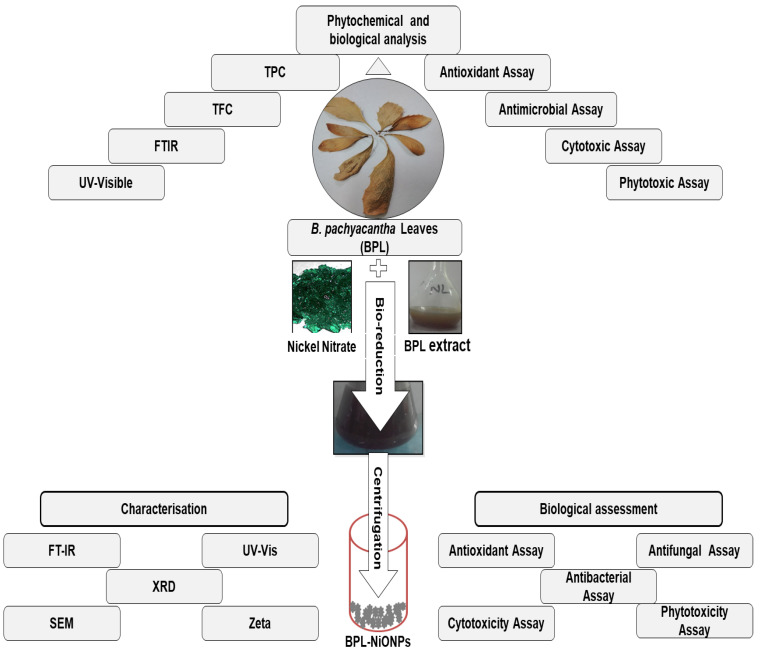
Layout of green synthesized BPL extract and BPL-NiONPs, characterizations and multiple biological applications.

The phytochemical capped NiONPs were analysed using different spectroscopic and microscopic techniques. The initial step of the reduction process was observed by visual and UV-Vis spectroscopy in the range of 200–800 nm. The role of phytochemicals as reducing, capping and stabilizing agents during the phytofabrication of BPL-NiONPs was characterized using Fourier transform infrared (FTIR) spectroscopy (Bruker FTIR spectrophotometer) in the range of 4000–400 cm^−1^, while the diffraction passions of BPL-induced NiONPs were examined by an X-ray diffractometer (XRD) (PANalytical XRD, The Netherlands). The elemental and morphological nature of prepared BPL-NiONPs were studied by an EDX spectrophotometer and SEM. Further, the stability and dispersity of BPL-NiONPs were checked by dynamic light scattering (DLS) using Malvern instrument (Malvern Zetasizer Malvern Panalytical LTD, EA Almelo, The Netherlands). Detailed information about BPL extract and BPL-NiONPs, characterizations and biological applications are provided in Figure 10.

### 4.3. Biological Analysis of BPL Extract and BPL Mediated NiONPs

#### 4.3.1. Total Phenolic and Total Flavonoid Contents Analysis (TPC and TFC)

Total phenolic contents in BPL extract and BPL-NiONPs were determined using Folin–Ciocalteu reagent [54]. In brief, 20 μL of the analysed samples were mixed with 90 μL of Folin–Ciocalteu reagent followed by 90 μL of NaCO_3_ solution. After incubation at room temperature for 60 min, absorbance was measured. Gallic acid was used as a reference for TPC.

Total flavonoids were estimated using the Aluminium Chloride Colorimetric method with some modification. Briefly, for the reaction mixture, 20 μL of BPL extract and BPL@NiONPs, 10 μL of aluminium chloride (10%), 10 μL of potassium acetate (1 M) and 160 μL of distilled water were mixed using a 96-well plate and subjected to incubation for 30 min. After incubation, absorbance of the solution was measured using a microplate reader at 405 nm. Total flavonoid contents in BPL extract and BPL@NiONPs were expressed as Quercetin equivalents (mg of QE/g) of the sample [55].

#### 4.3.2. 2,2-Diphenyl-1-picrylhydrazyl Assay (DPPH)

The free-radical-scavenging potential of BPL extract and synthesized BPL@NiONPs was evaluated by 2,2-diphenyl-1-picrylhydrazyl (DPPH) assay using a microplate reader [56]. For preparation of the reagent solution, 2.4 mg of DPPH was mixed with 25 mL of methanol. The procedure involved the addition of 180 μL of reagent solution into 20 μL of test sample to make the final 200 μL of reaction mixture. The mixture was subjected to shaking followed by incubation for 1 h. Further, absorbance was measured at 517 nm using a microplate reader to find radical scavenging activity using the formula below:

$$DPPH\;scavenging\;effect\;\%\;=\;\frac{AC-AS}{AC}\times 100$$

where AC and AS refer to absorbance of the control and sample at 517 nm.

#### 4.3.3. Total Antioxidant Capacity (TAC)

The antioxidant capacity of leaf extract was evaluated using the phosphomolybdenum method [56]. The reagent solutions of sulfuric acid (0.6 mol/L), sodium phosphate (28 mmol/L) and ammonium molybdate (4 mmol/L) were prepared. The tested sample (100 μL) was mixed with reagent solution (90 μL) and incubated at 95 °C for 90 min. The solution was cooled, and absorbance of the mixture was taken at 695 nm. Total antioxidant capacity was calculated as μg/mg equivalent of ascorbic acid.

#### 4.3.4. Antimicrobial Activities Using Agar Well Diffusion Method (AWDM)

The bacterial pathogens were provided and cultured by Plant Pathology Laboratory Quaid-i-Azam University, Islamabad, Pakistan. The agar well diffusion method was performed to study the antimicrobial activity of BPL extract and BPL-NiONPs against multidrug resistant (MDR) clinical isolates of *P. aeruginosa* (23451), *S. aureus* (48755) and *E. coli* (30155). The Muller–Hinton agar was prepared by suspending 34 g agar per litter in distilled water, adjusting the pH to 7, autoclaving for 15 min at 121 °C and finally cooling down to 50–45 °C. The media were poured into Petri plates (diameter = 14 cm). The wells were made and sealed by pouring 30 μL of liquid nutrient agar medium in each. Each well was filled using 100 μL of test solution. Kanamycin and distal water were used as positive and negative control, respectively. The plates were placed in the incubator for 24 h at 37 °C. After 24 h, zones of inhibition were measured.

#### 4.3.5. Antifungal Assay Using Poisoned Food Method (PFM)

Antifungal screening of BPL extract and BPL@NiONPs were determined using different phytopathogenic fungi such as *A. alternate*, *A. niger* and *F. oxysporum.* Sabouraud dextrose agar media (Oxoid CMO147) was prepared and autoclaved for the growth of fungal strains. Different concentrations (2000–50 μg/mL) of BPL extract and (1000–50 μg/mL) BPL@NiONPs were mixed with SDA (Sabouraud Dextrose Agar) medium and were shaken properly. A 5 mm in diameter disc of the 7-day-old culture of the above test fungus was placed at the centre of the Petri dish and incubated at 27 °C for 3 days and the growth was measured in millimetres. The SDA medium without plant extracts served as control and fluconazole was taken as positive control. The antifungal activity of tested samples in terms of percentage inhibition of mycelia growth were calculated using the formula below [57].

Percentage Inhibition = FC − FN/FC × 100

where FC and FN represent the average increase in fungal growth (F) in the control and each treatment (NPs).

#### 4.3.6. Cytotoxic Assay Using Brine Shrimp Lethality Assay (BSLA)

The cytotoxic effects of *B.* *Pachyancantha* leaf extract and BPL@NiONPs were evaluated using brine shrimp cytotoxicity assay. Artificial sea water was prepared by adding 3.8 g sea salt in 1 L distilled water using a partitioned hatching chamber. Eggs of brine shrimp (*Artemia* *salina)* were put in the covered portion of the chamber and incubated for 48 h at 30 °C. Four different concentrations of BPL extract and BPL@NiONPs (200–1 µg/mL) were added in each vial and their final volume was adjusted up to 5 mL by adding sea water. Then, ten mature brine shrimps were introduced into each vial and left under the lamp. After 24 h, alive brine shrimps were counted [58]. Potassium permanganate was used as a positive control while sea water and distal water were used as a negative control. Lethality concentration (LC_50_) values and percentage mortality were calculated.

#### 4.3.7. Phytotoxicity Assay Using Radish Seed Assay (RSA)

The phytotoxic effect of BPL extract and BPL-NiONPs were evaluated using the radish seed assay method. Different concentrations (1000–31.25 µg/mL) of tested samples were introduced in each Petri plate containing sterilized filter paper (Whatman filter paper). In each Petri plate, 15 seeds (after sterilization with sodium hypo chloride solution) were placed at room temperature in dim light. All plates were wrapped with parafilm and incubated at 25 °C in dim light. From day one to day five, seed germination indices were calculated [59,60].

#### 4.3.8. Statistical Analysis

The obtained numerical data were described in triplicate and enumerated as mean ± standard deviation. By one-way analysis of variance, the inhibitory activities of BPL@NiONPs on fungal, bacterial and seed germination were found. Homogeneousness of variance between means was assessed by Tukey’s test at a 95% confidence interval using Statistix version 10. The cytotoxic potential of BPL@NiONPs was estimated by finding the lethality concentration (IC_50_) values (GraphPad software, San Diego, CA, USA) [61,62].

## 5. Conclusions

In the current study, we introduced a sample, green, energy-efficient, eco-friendly and cost-effective method for the synthesis NiONPs using *Berberis pachyacantha* leaf extract. In the next step, the synthesis of BPL@NiONPs was confirmed using different characterization tools. The data revealed that synthesized BPL@NiONPs have a rhombohedral structure and are in the nano-scale range (22.53 nm). Once the synthesis was confirmed, the BPL@NiONPs were investigated for diverse in vitro bioactivities and revealed significant antibacterial, antifungal, antioxidant and cytotoxicity potential. More interestingly, at low concentrations, the prepared BPL-NiONPs speed up the seed germination as compared to non-treated seeds. This biofriendly NPs can be used for breaking and stimulating high dormancy, and thus help in seed germination. In future, we recommend different other in vitro and in vivo bioactivities to further study the biomedical applications of the *Berberis pachyacantha*-mediated nanoparticles.

## Figures and Tables

**Table 1:** FTIR analysis of leaf extracts of *B. pachyncantha*.

Peak Values (cm^−1^)	Strength	Functional Groups	Interpretations
**3278.91**	Medium	OH	Phenol, Alcohol
**2981.10–2840.51**	Medium	C-H	Alkane
**1645.87**	Medium	C=N	Imine
**1396.31**	Weak	C=C	Aromatic compounds
**1114.94–1013.15**	Weak	C–O	Alcohol, ether
**550.9**	Medium	C-Cl	Alkyl halides

(www.thermoscientific.com/ftir, accessed on 26 January 2022).

**Table 2:** XRD analysis of BPL based NiONPs.

No	2θ_B_	θ_B_	2Θ_1_	2Θ_2_	$\beta =\frac{2\theta 2-2\theta 1\;}{2}$ $\times \frac{\pi }{180}\;\;(\mathrm{Radian})$	Interplanar Spacing d (A°)	$D=\frac{0.9\;\lambda }{\beta \;cos\theta B}$ (nm)	Miller Indices (hkl)
1	37.04°	18.52°	36.75°	37.41°	0.00575	2.41200	24.7 nm	(003)
2	43.12°	21.56°	42.47°	43.53°	0.00925	2.08900	15.69 nm	(012)
3	62.77°	31.38°	62.45°	63.09°	0.00558	1.47700	28.33 nm	(104)

## Data Availability

Data are contained within the article.

## Sample Availability

Samples of the compounds are not available from the authors.

## References

[1] Iqbal J., Abbasi B.A., Munir A., Uddin S., Kanwal S., Mahmood T. (2020). Facile green synthesis approach for the production of chromium oxide nanoparticles and their different in vitro biological activities. Microsc. Res. Tech..

[2] Abbasi B.A., Iqbal J., Ahmad R., Zia L., Kanwal S., Mahmood T., Chen J.T. (2020). Bioactivities of Geranium wallichianum leaf extracts conjugated with zinc oxide nanoparticles. Biomolecules.

[3] Abbasi B.A., Iqbal J., Nasir J.A., Zahra S.A., Shahbaz A., Uddin S., Mahmood T. (2020). Environmentally friendly green approach for the fabrication of silver oxide nanoparticles: Characterization and diverse biomedical applications. Microsc. Res. Tech..

[4] Uddin S., Safdar L.B., Iqbal J., Yaseen T., Laila S., Anwar S., Quraishi U.M. (2021). Green synthesis of nickel oxide nanoparticles using leaf extract of Berberis balochistanica: Characterization, and diverse biological applications. Microsc. Res. Tech..

[5] Uddin S., Safdar L.B., Anwar S., Iqbal J., Laila S., Abbasi B.A., Quraishi U.M. (2021). Green synthesis of nickel oxide nanoparticles from Berberis balochistanica stem for investigating bioactivities. Molecules.

[6] Abbasi B.A., Iqbal J., Zahra S.A., Shahbaz A., Kanwal S., Rabbani A., Mahmood T. (2020). Bioinspired synthesis and activity characterization of iron oxide nanoparticles made using Rhamnus Triquetra leaf extract. Mater. Res. Express.

[7] Abbasi B.A., Iqbal J., Mahmood T., Ahmad R., Kanwal S., Afridi S. (2019). Plant-mediated synthesis of nickel oxide nanoparticles (NiO) via Geranium wallichianum: Characterization and different biological applications. Mater. Res. Express.

[8] Abbasi B.A., Iqbal J., Mahmood T., Qyyum A., Kanwal S. (2019). Biofabrication of iron oxide nanoparticles by leaf extract of Rhamnus virgata: Characterization and evaluation of cytotoxic, antimicrobial and antioxidant potentials. Appl. Organomet. Chem..

[9] Končić M.Z., Kremer D., Karlović K., Kosalec I. (2010). Evaluation of antioxidant activities and phenolic content of Berberis vulgaris L. and Berberis croatica Horvat. Food Chem. Toxicol..

[10] Hameed S., Iqbal J., Ali M., Khalil A.T., Abbasi B.A., Numan M., Shinwari Z.K. (2019). Green synthesis of zinc nanoparticles through plant extracts: Establishing a novel era in cancer theranostics. Mater. Res. Express.

[11] Bhatt L.R., Wagle B., Adhikari M., Bhusal S., Giri A., Bhattarai S. (2018). Antioxidant Activity, Total Phenolic and Flavonoid Content of Berberis aristata DC. and Berberis thomsoniana CK Schneid. from Sagarmatha National Park, Nepal. Pharmacog. J..

[12] Gundogdu M. (2013). Determination of antioxidant capacities and biochemical compounds of Berberis vulgaris L. fruits. Adv. Environ. Biol..

[13] Batool F., Saadullah M., Asif M., Uzair M., Choudhary B.A., Afzal S., Tareen R.B. (2019). Phytochemical and biological screening of root extracts of Berberis baluchistanica. BioCell.

[14] Baloch N., Nabi S., Yasser M., Kahraman A. (2013). In vitro antileishmanial, cytotoxic, anti-oxidant activities and phytochemical analysis of Berberis baluchistanica roots extracts and its fractions. J. Phytopharmacol..

[15] Belwal T., Bhatt I.D., Rawal R.S., Pande V. (2017). Microwave-assisted extraction (MAE) conditions using polynomial design for improving antioxidant phytochemicals in Berberis asiatica Roxb. ex DC. leaves. Ind. Crops Prod..

[16] Karimkhani M., Salarbashi D., Sefidy S.S., Mohammadzadeh A. (2019). Effect of extraction solvents on lipid peroxidation, antioxidant, antibacterial and antifungal activities of Berberis orthobotrys Bienerat ex CK Schneider. J. Food Meas. Charact..

[17] Elzaawely A.A., Xuan T.D., Koyama H., Tawata S. (2007). Antioxidant activity and contents of essential oil and phenolic compounds in flowers and seeds of Alpinia zerumbet (Pers.) BL Burtt. & RM Sm. Food Chem..

[18] Peterson J., Dwyer J. (1998). Flavonoids: Dietary occurrence and biochemical activity. Nutr. Res..

[19] WHO (2004). Vitamin and Mineral Requirements in Human Nutrition.

[20] Osawa T. (1994). Novel natural antioxidants for utilization in food and biological systems. Postharvest Biochem. Plant Food-Mater. Trop..

[21] Masek A., Latos-Brozio M., Chrzescijanska E., Podsedek A. (2019). Polyphenolic Profile and Antioxidant Activity of Juglans regia L. Leaves and Husk Extracts. Forests.

[22] Jain P., Soni A., Jain P., Bhawsar J. (2016). Phytochemical analysis of Mentha spicata plant extract using UV-VIS, FTIR and GC/MS technique. J. Chem. Pharmac. Res..

[23] Maobe M.A., Nyarango R.M. (2013). Fourier transformer infra-red spectrophotometer analysis of Warburgia ugandensis medicinal herb used for the treatment of diabetes, malaria and pneumonia in Kisii Region, Southwest Kenya. Glob. J. Pharmacol..

[24] Saxena S., Negi R., Guleri S. (2014). Antimicrobial potential of Berberis aristata DC against some human bacterial pathogens. Mycopathol. Res..

[25] Velioglu Y., Mazza G., Gao L., Oomah B. (1998). Antioxidant activity and total phenolics in selected fruits, vegetables, and grain products. J. Agric. Food Chem..

[26] Fernández-Poyatos M.d.P., Ruiz-Medina A., Zengin G., Llorent-Martínez E. (2019). Phenolic characterization, antioxidant activity, and enzyme inhibitory properties of Berberis thunbergii DC. leaves: A valuable source of phenolic acids. Molecules.

[27] Akhtar N., Mirza B. (2018). Phytochemical analysis and comprehensive evaluation of antimicrobial and antioxidant properties of 61 medicinal plant species. Arab. J. Chem..

[28] Malik A., Najda A., Bains A., Nurzyńska-Wierdak R., Chawla P. (2021). Characterization of citrusnobilis peel methanolic extract for antioxidant, antimicrobial, and anti-inflammatory activity. J. Mol..

[29] Bains A., Chawla P. (2020). In vitro bioactivity, antimicrobial and anti-inflammatory efficacy of modified solvent evaporation assisted Trametes versicolor extract. J. Biotech..

[30] Iqbal J., Abbasi B.A., Ahmad R., Mahmoodi M., Munir A., Zahra S.A., Shahbaz A., Shaukat M., Kanwal S., Uddin S. (2020). Phytogenic Synthesis of Nickel Oxide Nanoparticles (NiO) Using Fresh Leaves Extract of Rhamnus triquetra (Wall.) and Investigation of Its Multiple In Vitro Biological Potentials. Biomedicines.

[31] Iqbal J., Abbasi B.A., Mahmood T., Hameed S., Munir A., Kanwal S. (2019). Green synthesis and characterizations of Nickel oxide nanoparticles using leaf extract of Rhamnus virgata and their potential biological applications. Appl. Organomet. Chem..

[32] Abbasi B.A., Iqbal J., Khan Z., Ahmad R., Uddin S., Shahbaz A., Zahra S.A., Shaukat M., Kiran F., Kanwal S. (2021). Phytofabrication of cobalt oxide nanoparticles from Rhamnus virgata leaves extract and investigation of different bioactivities. Microsc. Res. Tech..

[33] Khalil A.T., Ovais M., Ullah I., Ali M., Shinwari Z.K., Hassan D., Maaza M. (2018). Sageretia thea (Osbeck.) modulated biosynthesis of NiO nanoparticles and their in vitro pharmacognostic, antioxidant and cytotoxic potential. Artif. Cells Nanomed. Biotechnol..

[34] Parthasarathy S., Jayacumar S., Chakraborty S., Soundararajan P., Joshi D., Gangwar K., Bhattacharjee A., Venkatesh M.P.D. (2020). Fabrication and characterization of copper nanoparticles by green synthesis approach using Plectranthus amboinicus leaves extract. Res. Seq..

[35] Abbasi B.A., Iqbal J., Kiran F., Ahmad R., Kanwal S., Munir A., Uddin S., Nasir J.A., Chalgham W., Mahmood T. (2020). Green formulation and chemical characterizations of Rhamnella gilgitica aqueous leaves extract conjugated NiONPs and their multiple therapeutic properties. J. Mol. Struct..

[36] Ghorbanpour M., Wani S.H. (2019). Advances in Phytonanotechnology: From Synthesis to Application.

[37] Jesudoss S., Vijaya J.J., Kennedy L.J., Rajan P.I., Al-Lohedan H.A., Ramalingam R.J., Kaviyarasu K., Bououdina M. (2016). Studies on the efficient dual performance of Mn1–xNixFe2O4 spinel nanoparticles in photodegradation and antibacterial activity. J. Photochem. Photobiol. Biol..

[38] Chawla P., Kumar N., Bains A., Dhull S.B., Kumar M., Kaushik R., Punia S. (2020). Gum arabic capped copper nanoparticles: Synthesis, characterization, and applications. Int. J. Biol. Macromol..

[39] Ali M., Haroon U., Khizar M., Chaudhary H.J., Hussain Munis M.F. (2021). Scanning electron microscopy of bio-fabricated Fe2O3 nanoparticles and their application to control brown rot of citrus. Microsc. Res. Tech..

[40] Khalil A.T., Ovais M., Ullah I., Ali M., Shinwari Z.K., Maaza M. (2020). Physical properties, biological applications and biocompatibility studies on biosynthesized single phase cobalt oxide (Co3O4) nanoparticles via Sageretia thea (Osbeck.). Arab. J. Chem..

[41] Huang W., Yan M., Duan H., Bi Y., Cheng X., Yu H. (2020). Synergistic antifungal activity of green synthesized silver nanoparticles and epoxiconazole against Setosphaeria turcica. J. Nanomat..

[42] Sabouri Z., Rangrazi A., Amiri M.S., Khatami M., Darroudi M.J.B., Engineering B. (2021). Green synthesis of nickel oxide nanoparticles using Salvia hispanica L.(chia) seeds extract and studies of their photocatalytic activity and cytotoxicity effects. J. Bioprocess Biosyst. Eng..

[43] Lin D., Xing B.J.E.p. (2007). Phytotoxicity of nanoparticles: Inhibition of seed germination and root growth. J. Environ. Poll..

[44] Szőllősi R., Molnár Á., Kondak S., Kolbert Z.J.P. (2020). Dual effect of nanomaterials on germination and seedling growth: Stimulation vs. phytotoxicity. J. Plants.

[45] Khalaki M.A., Moameri M., Lajayer B.A., Astatkie T. (2020). Influence of nano-priming on seed germination and plant growth of forage and medicinal plants. J. Plant Growth Regul.

[46] Maroufpoor N., Mousavi M., Hatami M., Rasoulnia A., Lajayer B.A. (2019). Mechanisms involved in stimulatory and toxicity effects of nanomaterials on seed germination and early seedling growth. Adva. Phytonanotechnol.

[47] Khan M.I., Fatima N., Shakil M., Tahir M.B., Riaz K.N., Rafique M., Iqbal T., Mahmood K. (2021). Investigation of in-vitro antibacterial and seed germination properties of green synthesized pure and nickel doped ZnO nanoparticles. Phys. B Condens. Matter.

[48] Younes N., Hassan H.S., Elkady M.F., Hamed A., Dawood M.F. (2020). Impact of synthesized metal oxide nanomaterials on seedlings production of three Solanaceae crops. Heliyon.

[49] F Elkady M., Shokry Hassan H. (2015). Equilibrium and dynamic profiles of azo dye sorption onto innovative nano-zinc oxide biocomposite. Curr. Nanosci..

[50] Hao Y., Zhang Z.-T., Rui Y.-K., Ren J.-Y., Hou T.-Q., Wu S.-J., Rui M.-M., Jiang F.-P., Liu L.-M. Effect of different nanoparticles on seed germination and seedling growth in rice. Proceedings of the 2nd Annual International Conference on Advanced Material Engineering (AME 2016).

[51] Chaudhary S., Kaur Y., Jayee B., Chaudhary G.R., Umar A. (2018). NiO nanodisks: Highly efficient visible-light driven photocatalyst, potential scaffold for seed germination of Vigna Radiata and antibacterial properties. J. Clean. Prod..

[52] Dawood M.F., Abeed A.H., Aldaby E.E. (2019). Titanium dioxide nanoparticles model growth kinetic traits of some wheat cultivars under different water regimes. Plant Physiol. Rep..

[53] Zulfiqar F., Navarro M., Ashraf M., Akram N.A., Munné-Bosch S. (2019). Nanofertilizer use for sustainable agriculture: Advantages and limitations. J. Plant Sci..

[54] Chlopicka J., Pasko P., Gorinstein S., Jedryas A., Zagrodzki P. (2012). Total phenolic and total flavonoid content, antioxidant activity and sensory evaluation of pseudocereal breads. Food Sci. Tech..

[55] Chang C.-C., Yang M.-H., Wen H.-M., Chern J.-C. (2002). Estimation of total flavonoid content in propolis by two complementary colorimetric methods. J. Food Drug Anal..

[56] Phull A.-R., Abbas Q., Ali A., Raza H., Zia M., Haq I.-u. (2016). Antioxidant, cytotoxic and antimicrobial activities of green synthesized silver nanoparticles from crude extract of Bergenia ciliata. Future J. Pharm. Sci..

[57] Singh J., Tripathi N. (1999). Inhibition of storage fungi of blackgram (*Vigna mungo* L.) by some essential oils. Flavour Fragr. J..

[58] Meyer B., Ferrigni N., Putnam J., Jacobsen L., Nichols D.J., McLaughlin J.L. (1982). Brine shrimp: A convenient general bioassay for active plant constituents. J. Planta Med..

[59] Rossello J.A., Mayol M. (2002). Seed germination and reproductive features of Lysimachia minoricensis (Primulaceae), a wildextinct plant. Ann. Bot..

[60] Hernández-Herrera R.M., Santacruz-Ruvalcaba F., Ruiz-López M.A., Norrie J., Hernández-Carmona G. (2014). Effect of liquid seaweed extracts on growth of tomato seedlings (*Solanum lycopersicum* L.). J. Appl. Phycol..

[61] Waghulde S., Kale M.K., Patil V. (2019). Brine Shrimp Lethality Assay of the Aqueous and Ethanolic Extracts of the Selected Species of Medicinal Plants. Multidiscip. Digit. Publ. Inst. Proc..

[62] Hayes A.W., Kruger C.L. (2014). Hayes’ Principles and Methods of Toxicology.

